# Fucose ameliorates the proinflammatory property of *Fusobacterium nucleatum* in colitis via altering its metabolism

**DOI:** 10.3389/fcimb.2023.1190602

**Published:** 2023-05-01

**Authors:** Caihan Duan, Lingzhi Hou, Xiaohua Deng, Junhao Wu, Wei Qian, Chaoqun Han, Xiaohua Hou

**Affiliations:** ^1^ Division of Gastroenterology, Union Hospital, Tongji Medical College, Huazhong University of Science and Technology, Wuhan, China; ^2^ Hubei Center of Industrial Culture Collection and Research, Wuhan, China

**Keywords:** *Fusobacterium nucleatum*, inflammatory bowel disease, fucose, metabolism, intestinal epithelial cell

## Abstract

**Introduction:**

Previous studies reported that fucose plays a protective role in inhibiting pathogens. Fusobacterium nucleatum (Fn) was recently found to promote the progression of colitis. However, the effects of fucose on Fn are poorly understood. This study aimed to explore whether fucose could ameliorate the proinflammatory property of Fn in colitis and the underlying mechanisms.

**Methods:**

To validate our hypothesis, mice were administrated with Fn and fucose-treated Fn (Fnf) before dextran sulfate sodium (DSS) treatment to establish Fn related colitis model. The metabolism variation of Fn was detected by metabolomic analysis. To verify the effects of bacterial metabolites on intestinal epithelial cells (IECs), Caco-2 cells were treated with bacterial supernatant.

**Results:**

More severe inflammation, intestinal barrier damage, autophagy block, and apoptosis in the colon were noted in DSS mice that were administrated with Fn or Fnf. However, the severity degree in Fnf+DSS group was less compared to Fn+DSS group. Metabolic pathways of Fn were altered after fucose treatment and proinflammatory metabolites were decreased. The supernatant of Fnf induced a lower level of inflammation than Fn in Caco-2 cells. One of the decreased metabolites, homocysteine thiolactone (HT), was proven to induce inflammatory effects in Caco-2 cells.

**Discussion:**

In conclusion, fucose ameliorates the proinflammatory property of Fn via altering its metabolism and these findings provide evidence for the application of fucose as functional food or prebiotic in the treatment of Fn related colitis.

## 1 Introduction

Inflammatory bowel disease (IBD) is a remittent and progressive inflammatory gastrointestinal disorder that causes a large global burden (Kaplan, 2015). Though the exact pathogenesis of IBD remains unclear, it is widely accepted that compositional and metabolic changes in the intestinal microbiota are closely associated with IBD (Lee and Chang, 2021; Michaels and Madsen, 2023). However, the relationship between IBD and dysbiosis still needs to be further explored.

*Fusobacterium nucleatum* (*Fn*), a commensal bacterium with potential pathogenicity, attracted many concerns in recent years. Researchers found a correlation between *Fn* and clinical features of IBD at first. About two-thirds of *Fusobacterium* spp. isolated from patients with the gastrointestinal disease were identified as *Fn* while *Fn* strains recovered from inflamed tissue showed stronger invasive ability on intestinal epithelium cell lines than those from healthy tissue. In addition, *Fusobacterium* is associated with the persistence of colonic inflammation in IBD and seems to consequently contribute to the pathogenesis of colorectal cancer (Strauss et al., 2011; Tahara et al., 2015; Bashir et al., 2016). Whereafter more and more research demonstrated through experiments that *Fn* could exacerbate colitis. Recent studies found that *Fn* regulates M1 macrophage polarization, promotes expression of inflammatory cytokines, and induces autophagy disorder, oxidative stress damage, and epithelial cell death, thus leading to intestinal epithelial barrier damage and aggravating colitis (Liu et al., 2019; Chen et al., 2020; Duan et al., 2021; Liu et al., 2021). Therefore, exploring the virulence factors of *Fn* and the corresponding treatment is worth considering in *Fn*-related colitis.

Fucose is involved in maintaining of gut homeostasis since the α1,2-fucosyl glycans expressed on intestinal epithelial cells work as a biological interface for the host-microbe interaction (Goto et al., 2016). We previously discovered that exogenous fucose could protect the intestinal mucosal barrier and alleviate dextran sulfate sodium (DSS) induced colitis (Li et al., 2021). More importantly, fucose could improve gut microbiome dysbiosis and regulate bile acid metabolism in colitis (Ke et al., 2020). It is reported that fucose was metabolized by gut microbiota and affects their metabolic pathways and the expression of virulence genes (Pickard et al., 2014). Since there are correlations between bacterial metabolism and virulence, and virulence gene regulators are affected by changes in carbon source availability (Poncet et al., 2009), we assumed that fucose may mitigate the proinflammatory property of *Fn* by affecting its metabolism.

To verify this hypothesis, we administrated mice that received DSS treatment (or not) with *Fn* or fucose-treated *Fn* in the current study to investigate their proinflammatory characteristic. Metabolomics analysis was adopted to explore the effects of fucose on the metabolism of *Fn*. Furthermore, bacterial supernatant of *Fn* and fucose-treated *Fn* was used to treat intestinal epithelial cell line to detect whether the altered metabolites of *Fn* cause less damage to intestinal epithelial cells (IECs). Our results revealed that fucose could ameliorate the proinflammatory property of *Fn* via altering its metabolism.

## 2 Materials and methods

### 2.1 Cell and bacterial strain

Human epithelial colorectal adenocarcinoma Caco-2 cells were cultured in Roswell Park Memorial Institute (RPMI) 1640 medium (Gibco; Thermo Fisher Scientific, Inc.) supplemented with 10% fetal bovine serum (Gibco; Thermo Fisher Scientific, Inc.) and 100 U/ml streptomycin/penicillin (Gibco; Thermo Fisher Scientific, Inc.) at 37˚C under 5% CO2.

*Fusobacterium nucleatum* (ATCC 25586) was purchased from Wuhan Research Institute of First Light Industry (Wuhan, China) and cultured in brain heart infusion (BHI) culture medium (pH 7.4). The methods of bacterial culture were as previously described (Lin et al., 2020). To investigate the effect of L-fucose on the pro-inflammatory properties of *Fn*, 0.5% fucose (Sigma, F2252) was added to the bacterial culture medium (*Fnf*). Caco-2 cells were treated with supernatant of *Fn* or *Fnf* to investigate the effect of bacterial metabolites.

### 2.2 Animal models

Mice were housed in the specific pathogen-free (SPF) grade facility of Huazhong University of Science and Technology and maintained at 12 h light/dark cycles with free access to food and water. All animal experiments in this study were approved by the Animal Experimentation Ethics Committee of Huazhong University of Science and Technology (Approval ID 2020-2529) and performed following national and EU guidelines.

To investigate if there were differences in the effects of *Fn* and *Fnf* on colitis, adult male mice (8 weeks) were randomly divided into 6 groups: Control, *Fn*, *Fnf*, DSS, DSS+*Fn*, and DSS+*Fnf* (4 mice/group in Control, *Fn* and *Fnf* groups and 8 mice/group in DSS, DSS+*Fn* and DSS+*Fnf* groups). Daily gavage of 10^9^ CFU *Fn* or *Fnf* phosphate- buffered saline (PBS) solution was conducted 7 days before DSS administration as previously described (Liu et al., 2019) and colitis was induced by 3% (wt/vol) DSS (MP Biomedicals, 160110) in the drinking water for 7 days. Control mice were given PBS and standard laboratory drinking water, respectively. The DSS group mice received PBS administration before DSS treatment. The DSS solution was refreshed every 2 days and the leftover DSS solution was measured. Mice were sacrificed on day 8. Tissues and blood were collected for subsequent analysis. Anesthetization was conducted before sacrificed by intraperitoneal injection of 50 mg/kg pentobarbital. Body weight, stool consistency, and any bleeding were examined every day to monitor disease activity index (DAI), which was calculated as previously described (Cooper et al., 1993). In brief, weight loss was calculated as: 0, no loss; 1, 1−5%; 2, 5−10%; 3, 10−20% and 4, >20%; stool consistency was calculated as: 0, normal; 2, loose stool; 4, diarrhea and stool bleeding were calculated as: 0, no blood; 2, presence and 4, gross blood.

### 2.3 Histological examination

For histological analysis, distal colon specimens were fixed in 4% formalin for 24 h and embedded in paraffin. 4μm thick sections were stained with hematoxylin and eosin (HE) and analyzed by a pathologist without prior knowledge of experimental design. Histological analysis was performed as previously described (Horino et al., 2008) based on inflammation severity (0, none; 1, mild; 2, moderate; 3, severe), inflammation extent (0, none; 1, mucosa; 2, mucosa and sub-mucosa; 3, transmural) and crypt damage (0, none; 1, basal 1/3 damaged; 2, basal 2/3 damaged; 3, crypts lost and surface epithelium present; 4, crypts and surface epithelium lost).

### 2.4 Immunofluorescence

For tissue immunofluorescence, paraffin-embedded colon tissues sections (4μm) were deparaffinized, hydrated, and treated with citrate buffer (pH 6.0) for antigen retrieval and then treated with 0.3% Triton for 15 min and blocked with 10% donkey serum for 1 h at room temperature. Tissues were incubated with ZO-1 (Invitrogen, 61-7300), occludin (Invitrogen, 42-2400), cladudin1 (GeneTex, GTX54539), LC3B (Cell Signaling Technology, 43566) and p62 (GeneTex, GTX100685) primary antibodies (all 1:200 dilution) overnight under 4°C. After washing with PBS 3 times, tissues were incubated with corresponding secondary antibodies conjugated Alexa Fluor 488 or 594 (Antgene, ANT024S and ANT030S) for 1 h at room temperature and then stained with DAPI (Antgene, ANT165) for 5 min for nuclear staining. For cell immunofluorescence, the slides of cells were fixed with 4% formalin for 30 min and subsequent works were similar to tissues from the 0.3% Triton treatment step. The images were acquired by a confocal laser microscope (Nikon).

### 2.5 Western blot analysis

Tissue or cell proteins were harvested with RIPA Lysis Buffer (Beyotime, P0013B) supplemented with phenylmethyl sulfonyl fluoride (PMSF, Beyotime ST506) protease inhibitor and phosphatase inhibitor. Total protein concentration was measured by BCA Protein Assay Kit (Thermo Fisher Scientific, 23225). Total proteins were separated by sodium dodecyl sulfate polyacrylamide gel electrophoresis (SDS−PAGE) and transferred to a PVDF membrane, followed by blocking with 5% skimmed milk for 1 h. Then the membranes were incubated with ZO-1 (Invitrogen, 61-7300), occludin (Invitrogen, 42-2400), cladudin1 (GeneTex, GTX54539), LC3B (Cell Signaling Technology, 43566), p62 (GeneTex, GTX100685), caspase3 (Cell Signaling Technology, 9662), cleaved-caspase3 (Cell Signaling Technology, 9664), Bcl2 (Proteintech, 12789-1-AP), Bax (Proteintech, 50599-2-Ig), GAPDH (ABclonal, A19056) and ACTB (ABclonal, AC038) primary antibodies (all 1:1000 dilution) overnight under 4°C. After washing with TBST for 3 times, membranes were incubated with corresponding secondary antibodies conjugated HRP (Antgene, ANT020 and ANT019) for 1 h at room temperature. Protein bands were visualized by the Chemi-luminescence imaging system (UVP, USA) using enhanced chemiluminescent reagents (Beyotime, P0018).

### 2.6 TUNEL assays

One Step TUNEL Apoptosis Assay Kit (Beyotime, C1088) was used for detecting cell apoptosis following the manufacturer’s instructions. Briefly, paraffin-embedded colon tissue sections were deparaffinized, hydrated and treated with protease K (Beyotime, ST535) for 15 min at room temperature. After washing 3 times with PBS, the sections were incubated with TUNEL dilution including TdT and fluorescein-dUTP for 1 h at 37°C. DAPI was used for nuclear staining.

### 2.7 RNA extraction and qPCR

Total RNA was extracted from tissues or cells using TRIzol reagent (Invitrogen, 15596018) according to the manufacturer’s instructions and reversed to cDNA using Prime Script RT Master Mix (Takara Biotechnology, RR036). qPCR was performed using the LightCycler® 480 SYBR I Master Mix (Roche Diagnostics), running on a Roche LightCycle R480 system (Roche Diagnostics). The relative fold change of mRNA expression was normalized relative to GAPDH and measured by the 2^−ΔCT^ method. The primer sequences are presented in **Table 1**.

**Table 1:** Sequences of primers used for RT-qPCR.

Gene	Forward primer (5’-3’)	Reverse primer (5’-3’)
Human genes		
IL-6	ATGAGGAGACTTGCCTGGTG	GGCATTTGTGGTTGGGTCAG
IL-8	CACTGCGCCAACACAGAAAT	AACTTCTCCACAACCCTCTGC
TNF-α	TACTCCCAGGTCCTCTTCAAGG	TTGATGGCAGAGAGGAGGTTG
GAPDH	ACCCACTCCTCCACCTTTGA	AAAGTGGTCGTTGAGGGCAA
Mouse genes		
IL-1β	CTGAACTCAACTGTGAAATGCC	CTTGTTGATGTGCTGCTGCG
IL-6	ACAAAGCCAGAGTCCTTCAGAG	CCACTCCTTCTGTGACTCCA
TNF-α	ACCCTCACACTCACAAACCAC	TAGCAAATCGGCTGACGGTG
GAPDH	CATGGCCTTCCGTGTTCCTA	TACTTGGCAGGTTTCTCCAGG

### 2.8 Transepithelial electrical resistance measurement

For TEER measurement, Caco-2 cells were plated into upper inserts of transwell chamber (1.12 cm^2^ area, 0.4 μm pore size; Corning) at a density of 10^5^ cells/well and cultured for 2 weeks to form polarized confluent monolayers. TEER was measured by EVOM TEER meter (World Precision Instruments) to evaluate the barrier functions of the monolayers. Measurements were performed every 6 hours after cells were treated with bacterial supernatant.

### 2.9 Cell apoptosis detection by flow cytometry

PI-Annexin V/FITC apoptosis detection kit (AntGene, ANT002) was used to examine Caco-2 cell apoptosis after being treated with bacterial supernatant according to the manufacturer’s instructions. Briefly, cells were collected from the culture plate using trypsin without EDTA, washing with PBS, then incubated with Annexin V-FITC and Propidium Iodide (PI) for 10 min at room temperature and analyzed with a FACS (BD FACSCanto) at Ex/Em: 488/519nm for Annexin V-FITC and 538/617nm for PI. The results were analyzed using FlowJo software.

### 2.10 mCherry-EGFP-LC3 adeno-associated virus transfection

To monitor autophagic flux, mCherry-EGFP-LC3 AAV (HANBIO) was transfected to Caco-2 cells. When cells grew to 50−70% confluence, cells were transfected with adenovirus (MOI=200) for 2 h. Then replacing the medium. Bacterial supernatant treatments were performed on the next day. Cells were fixed with 4% formalin and observed under a confocal laser scanning microscope (Olympus) 48 h after transfection.

### 2.11 Transmission electron microscopy

Cells were harvested after treatment with bacterial supernatant and fixed with 2.5% glutaraldehyde in PBS, post−fixed in 1% osmium tetroxide for 1 h, rinsed with 0.1 M phosphate buffer (pH 7.4), dehydrated with increasingly graded alcohols before being embedded in Epon. Ultrathin sections were cut using an ultramicrotome and examined by FEI Tecnai G2 12TEM (FEI Company).

### 2.12 Metabolomics analysis

Bacterial supernatant samples were processed and untargeted metabolic profiling of bacterial supernatant was performed by liquid chromatography-mass spectrometry (LC-MS) as described previously (Zou et al., 2013). Briefly, metabolite samples were extracted from the supernatant with methanol and acetonitrile combined with isotope-labeled compounds. Samples were detected by Vanquish Ultra-high performance liquid chromatograph (Thermo Fisher Scientific) and separated by Waters ACQUITY UPLC BEH Amide liquid chromatography. Data were collected with a Thermo Q Exactive HFX mass spectrometer (Thermo Fisher Scientific). Original data was managed as follows: removing deviation value and missing value, filling missing value, and normalization (Dunn et al., 2011). Then performing hierarchical clustering analysis and principal component analysis (Ringnér, 2008). Differential metabolites were screened through variable importance in projection (VIP) value combined with P value and fold change. Kyoto Encyclopedia of Genes and Genomes (KEGG) pathway database was used for annotation of the different metabolites as described previously (Ogata et al., 1999).

### 2.13 Statistical analysis

Experiments data were analyzed using SPSS Statistics 25.0, GraphPad Prism 7.0, nd ImageJ and presented by GraphPad Prism 7.0. All experiments were performed at least in triplicate and values are shown as means ± SEM. Significant differences were calculated using Student’s two-tailed t-test between 2 groups and one−way ANOVA among multiple groups. Statistical significance was defined at P<0.05.

## 3 Results

### 3.1 Fucose intervention mitigate the proinflammatory property of *Fn* in DSS-induced colitis

**Figure 1:**
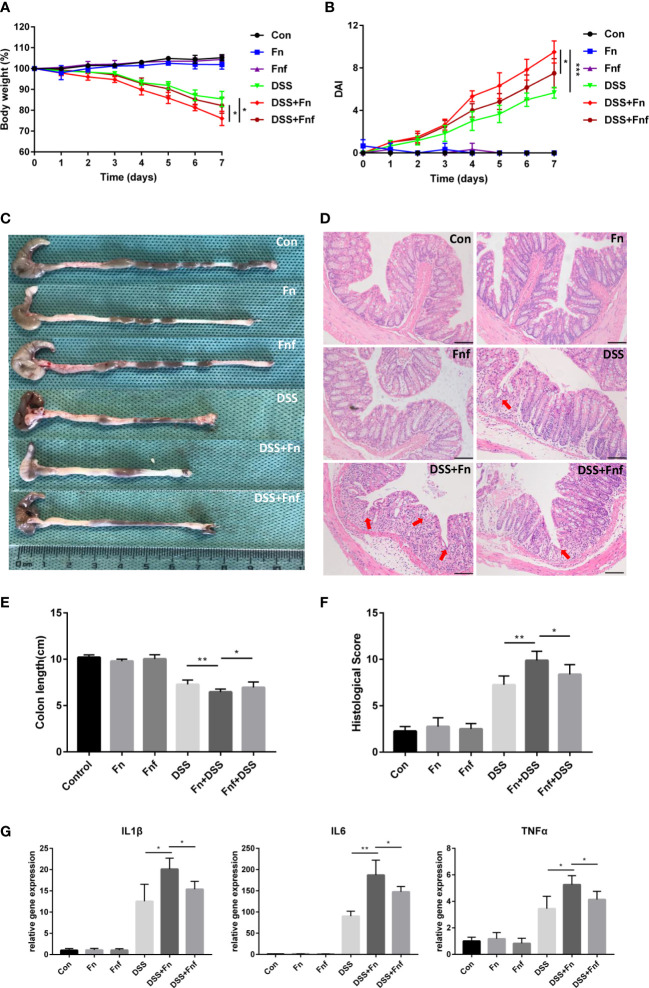
Fucose intervention mitigate the proinflammatory property of *Fn* in DSS-induced colitis. **(A)** Body weight changes during the acute colitis process. **(B)** DAI evaluation during the acute colitis process. **(C)** Colon length of mice in each group. **(D)** HE staining of sections from colon tissues, the arrows indicate epithelial structure lost (Scale bar, 100 μm). **(E)** statistical analysis of colon length. **(F)** Histopathological scores of colon tissues in each group. **(G)** mRNA expression of inflammatory cytokines in colon tissues. *P<0.05, **P<0.01, ***P<0.001.

Considering that *Fn* could contribute to the progression of colitis, we treated DSS-induced colitis mice with *Fn* and *Fnf* to determine the effects of fucose on the proinflammatory property of *Fn*. Single *Fn* or *Fnf* administration showed no significant influence on mice in the process of model establishment. However, after administrated with these two kinds of bacteria (*Fn* and *Fnf*), mice showed more severe body weight loss in the process of DSS treatment while the body weights of DSS+*Fnf* group mice were higher than that in DSS+*Fn* group (**Figure 1A**). Also, *Fn* treatment induces more severe symptoms in DSS mice than *Fnf*, which was indicated by the DAI (**Figure 1B**). Along with these, *Fn* treatment resulted in decreased colon length in DSS mice compared to *Fnf* (**Figures 1C, E**). Through HE staining, we could also find more severe mucosal ulceration, inflammatory cell infiltration, crypt, and gland destruction in DSS+*Fn* group mice than DSS+*Fnf* group mice (**Figures 1D, F**). Moreover, qPCR showed that the mRNA expression level of inflammatory cytokines IL-1β, IL-6, and TNF-α was higher in DSS+*Fn* group compared to DSS+*Fnf* group (**Figure 1G**). All these results demonstrated that the proinflammatory property of *Fn* in the colon was weakened by fucose treatment.

### 3.2 Fucose-treated *Fn* caused less intestinal tight junction damage in colitis

**Figure 2:**
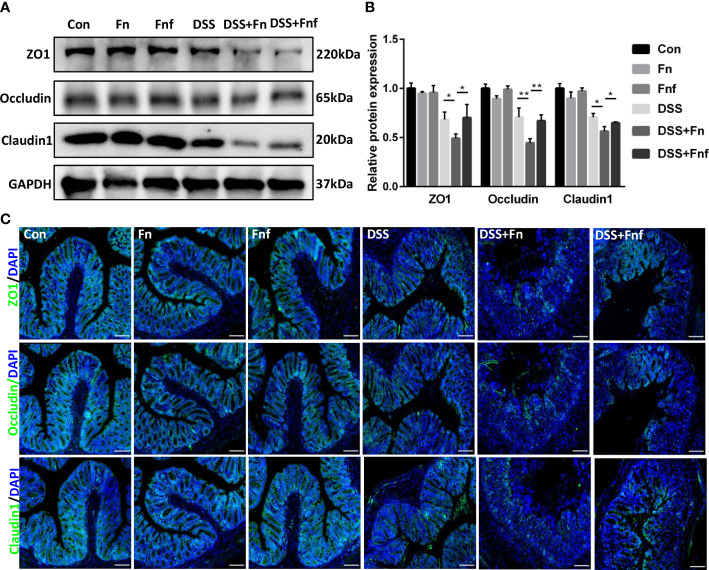
Fucose-treated *Fn* caused less intestinal tight junction damage in colitis. **(A, B)** Representative ZO-1, occludin and claudin1 western blots of colon tissues and statistical analysis. **(C)** Representative immunofluorescence images of ZO-1, occludin and claudin1 in colon tissues (Scale bar, 100 μm). *P<0.05, **P<0.01.

Intestinal epithelial tight junction maintains the intestinal epithelial barrier. *Fn* was reported to damage epithelial integrity via destructing tight junction (Liu et al., 2020). Therefore, we examined the tight junction in the *Fn* and *Fnf*-related DSS-induced colitis. Western blot analysis showed that tight junction proteins ZO-1, occluding, and claudin1 expression in DSS+*Fn* group was lower compared to DSS only group (**Figures 2A, B**), while the expression in DSS+*Fnf* group was higher than DSS+*Fn* group (**Figures 2A, B**), and the tendency in immunofluorescence experiments was similar (**Figure 2C**). These results indicated that *Fnf* caused less damage to the intestinal tight junction compared to *Fn*.

### 3.3 Fucose-treated *Fn* caused less autophagy block and apoptosis in IECs

**Figure 3:**
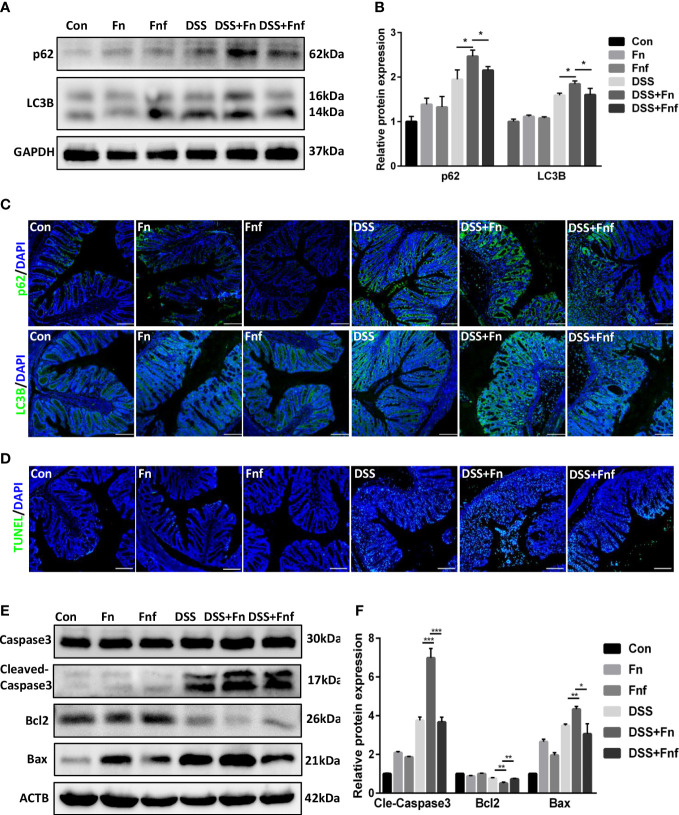
Fucose-treated *Fn* caused less autophagy block and apoptosis in IECs. **(A, B)** Representative LC3B and SQSTM1/p62 western blots of colon tissues in each group and statistical analysis. **(C)** Representative immunofluorescence images of LC3B and SQSTM1/p62 in colon tissues (Scale bar, 100 μm). **(D)** Representative TUNEL images of colon tissues (Scale bar, 100 μm). **(E, F)** Representative caspase3, cleaved-caspase3, bcl2 and bax western blots of colon tissues in each group and statistical analysis. *P<0.05, **P<0.01, ***P<0.001.

We previously found that *Fn* exacerbates colitis through inhibiting autophagic flux (Duan et al., 2021), thus we analyzed the level of autophagy in the IECs here. As shown in **Figures 3A, B**, though the expression level of autophagy marker LC3B-II in *Fn-*infected mice was higher than *Fnf* infected mice, the p62 expression was increased at the same time, which indicated that the autophagy was blocked more severely in the former. This was further demonstrated by immunofluorescence analysis (**Figure 3C**).

Moreover, intestinal epithelial apoptosis was related to IBD pathogenesis. It was confirmed that autophagy could protect IECs from cytokine-induced apoptosis (Larabi et al., 2020). Therefore, we supposed that *Fn* infection resulted in more severe epithelial apoptosis. TUNEL assays showed that *Fn* treatment led to more severe epithelial apoptosis in DSS mice, while there were fewer apoptotic cells in *Fnf*+DSS group (**Figure 3D**). We further examined the expression of apoptosis markers bcl2, bax, and caspase3. Results showed that the expressions of bax and cleaved-caspase3 were obviously increased in *Fn*+DSS group compared to *Fnf*+DSS group while the expression of bcl2 was decreased, indicating activated apoptosis (**Figures 3E, F**).

### 3.4 Fucose treatment altered the metabolism of *Fn*

**Figure 4:**
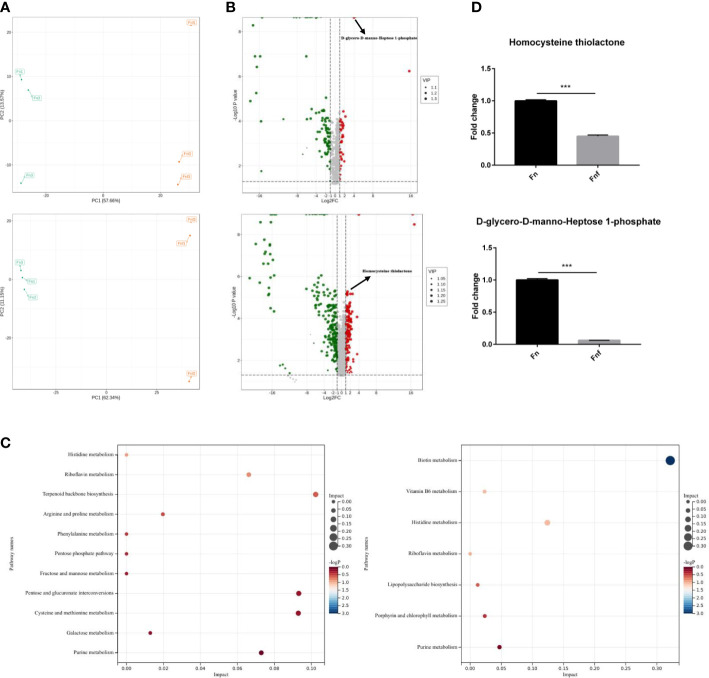
Fucose treatment altered metabolism of *Fn*. **(A)** Principal component analysis of metabolites of *Fn* and fucose treated *Fn*. **(B)** Volcano plot of different expressed metabolites. **(C)** KEGG annotation of the different metabolites. **(D)** Fold change of D-glycero-D-manno-Heptose 1-phosphate and HT in the two groups. ***P<0.001.

Since fucose treatment could alter bacterial metabolism as described above, we detected the metabolites of *Fn* and *Fnf* through untargeted metabolomics analysis performed by LC-MS. Principal component analysis (PCA) showed that there was a significant separation between *Fn* and *Fnf* groups under positive ion mode as well as negative ion mode (**Figure 4A**). There were 48 upregulated metabolites and 190 downregulated metabolites in *Fn* group compared to *Fnf* under positive ion mode, while 240 upregulated metabolites and 382 downregulated metabolites under negative ion mode, as shown by the volcano plot (**Figure 4B**). KEGG annotation of differential metabolites revealed that biotin metabolism, vitamin B6 metabolism, histidine metabolism, lipopolysaccharide biosynthesis, and terpenoid backbone biosynthesis were enhanced in *Fn* group (**Figure 4C**). In addition, we found that D-glycero-D-manno-Heptose 1-phosphate, which was involved in lipopolysaccharide (LPS) synthesis, and homocysteine thiolactone (HT), the cyclic thioester of homocysteine, were decreased in *Fnf* supernatant (**Figure 4D**). Both of these two metabolites were related to inflammatory disorders (Adekoya et al., 2018; AnandBabu et al., 2019). Collectively, these data indicate that fucose may ameliorate the proinflammatory property of *Fn* by altering its metabolism.

### 3.5 The supernatant of fucose-treated *Fn* caused less damage to Caco-2 monolayer

**Figure 5:**
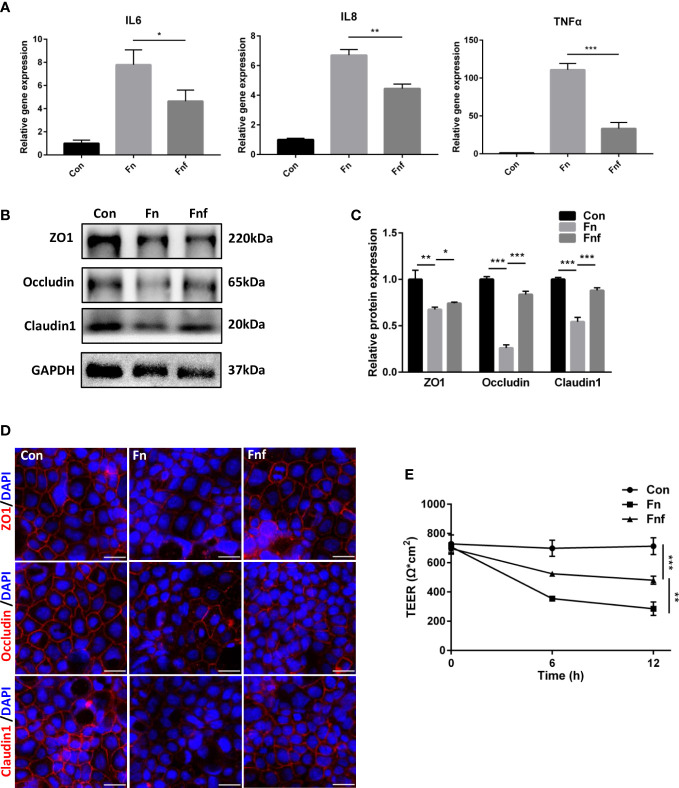
The supernatant of fucose-treated *Fn* caused less damage to Caco-2 monolayer. **(A)** Relative gene expression of IL-6, IL-8 and TNF-α in Caco-2 cells treated with supernatant of *Fn* and *Fnf*. **(B, C)** Representative ZO-1, occludin and claudin1 western blots of Caco-2 cells and statistical analysis. **(D)** Representative immunofluorescence images of ZO-1, occludin and claudin1 in Caco-2 cells (Scale bar, 50 μm). **(E)** TEER measurement of Caco-2 monolayer treated with *Fn* and *Fnf*. *P<0.05, **P<0.01, ***P<0.001.

To further explore whether the fucose-induced metabolism alteration alleviate the proinflammatory property of *Fn*, we treated Caco-2 cells with supernatant of *Fn* and *Fnf*. As expected, mRNA expression of inflammation cytokines IL-6, IL-8, and TNF-α in *Fn-*treated cells was higher than *Fnf* (**Figure 5A**). The expression of tight junction proteins ZO-1, occludin, and claudin1 was declined when *Fn* or *Fnf* was added. However, the reduction was more obvious after *Fn* treatment compared to *Fnf* (**Figures 5B, C**). The fluorescence intensity variation of these three proteins was consistent with western blot results (**Figure 5D**). Moreover, TEER measurement showed that transepithelial electrical resistance of Caco-2 monolayer was constantly decreasing after bacterial supernatant was added. And the value was lower in *Fn* group than *Fnf* (**Figure 5E**). These results indicated that the fucose treatment restrain the *Fn*-induced impairment in Caco-2 monolayer.

### 3.6 Supernatant of fucose-treated *Fn* caused milder autophagy block and apoptosis in Caco-2 cells

**Figure 6:**
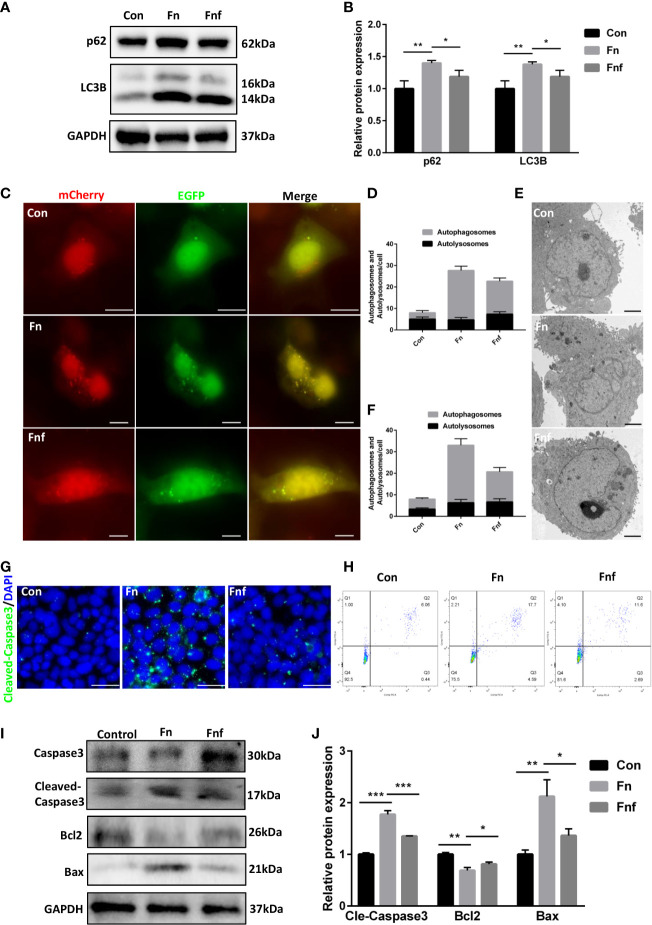
Supernatant of fucose-treated *Fn* caused milder autophagy block and apoptosis in Caco-2 cells. **(A, B)** Representative LC3B and SQSTM1/p62 western blots of Caco-2 cells and statistical analysis. **(C)** Confocal micrographs of mRFP-GFP-LC3 in Caco-2 cells treated with *Fn* and *Fnf* (Scale bar, 10 μm). **(D)** TEM images of Caco-2 cells illustrating the autophagosomes and autolysosomes (Scale bar, 2 μm). **(E, F)** Statistical analysis of autophagosomes and autolysosomes. **(G)** Representative immunofluorescence images of cleaved-caspase3 in Caco-2 cells (Scale bar, 50 μm). **(H)** Flow cytometry analysis of apoptosis in Caco-2 cells by Annexin V-FITC and PI. **(I, J)** Representative caspase3, cleaved-caspase3, bcl2 and bax western blots of Caco-2 cells and statistical analysis. *P<0.05, **P<0.01, ***P<0.001.

We analyzed the autophagy and apoptosis level in Caco-2 cells to further verify the effects of altered metabolites. After being treated with *Fn*, expression of LC3B-II and p62 was increased in Caco-2, while *Fnf* treatment could not exert the same level of effects (**Figures 6A, B**). Furthermore, we detected autophagosomes and autolysosomes in Caco-2 cells through mRFP-GFP-LC3 adenovirus transfection and TEM. As shown in **Figures 6C, D**, there were fewer yellow dots, which indicate autophagosomes and autophagy block, in *Fnf-*treated cells compared to *Fn* treated. This can be proved by TEM observation, which showed a higher autophagosome to autolysosome ratio in the *Fn* group (**Figures 6E, F**). In addition, we found that there were fewer cleaved-caspase 3 positive cells in *Fnf* group than *Fn* group (**Figure 6G**). Flow cytometry analysis showed that the total number of Annexin V+/PI+ (indicating apoptotic cells at late stage) and Annexin V+/PI- cells (indicating apoptotic cells at early stage) increased less after *Fnf* intervention compared to *Fn* (**Figure 6H**). Consistent with these, there was higher protein expression of bax and cleaved-caspase3 and lower expression of bcl2 in *Fn*-treated Caco-2 than *Fnf* treated (**Figures 6I, J**). All these results demonstrated that the fucose-altered metabolites of *Fn* caused milder autophagy block and apoptosis in Caco-2 cells.

**Figure 7:**
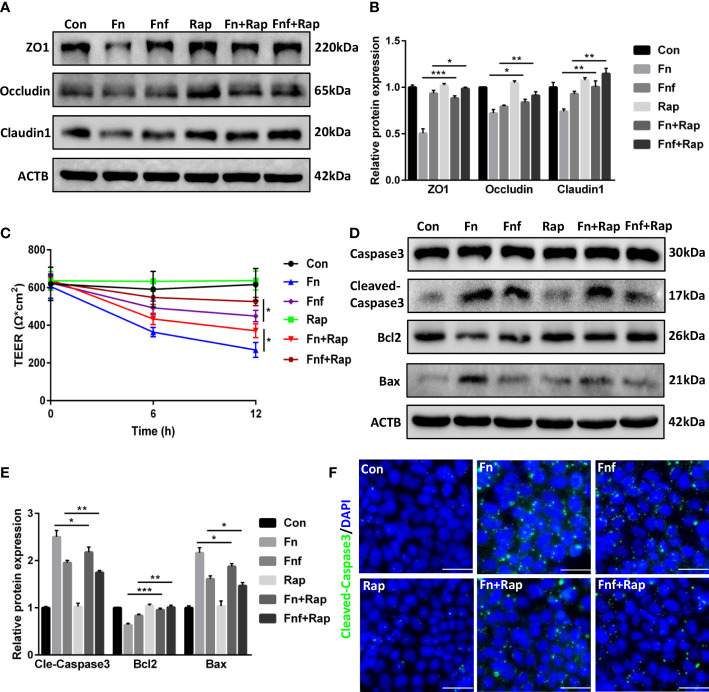
Autophagy activator alleviated *Fn* and *Fnf* induced tight junction damage and apoptosis in Caco-2 cells **(A, B)** Representative LC3B and SQSTM1/p62 western blots of Caco-2 cells treated with *Fn*, *Fnf* and rapamycin and statistical analysis. **(C)** TEER measurement of Caco-2 monolayer treated with *Fn*, *Fnf* and rapamycin. **(D, E)** Representative caspase3, cleaved-caspase3, bcl2 and bax western blots of Caco-2 cells and statistical analysis. **(F)** Representative immunofluorescence images of cleaved-caspase3 in Caco-2 cells (Scale bar, 50 μm). *P<0.05, **P<0.01, ***P<0.001.

To further confirm the role of autophagy in the proinflammatory property of *Fn* and *Fnf*, we added autophagy activator rapamycin at the meanwhile of bacterial supernatant treatment. Western blot analysis showed that rapamycin improved the tight junction proteins expression to a certain extent (**Figures 7A, B**). TEER measurement showed an increase in resistance of monolayer (**Figure 7C**), indicating that activating autophagy could partly restore the tight junction injury. Also, we tested the expression of apoptosis-related proteins and found there was an improvement in expression of bax, cleaved-caspase3, and bcl2 under the effect of rapamycin (**Figures 7D–F**).

### 3.7 Homocysteine thiolactone may play a role in the proinflammatory property of *Fn*

**Figure 8:**
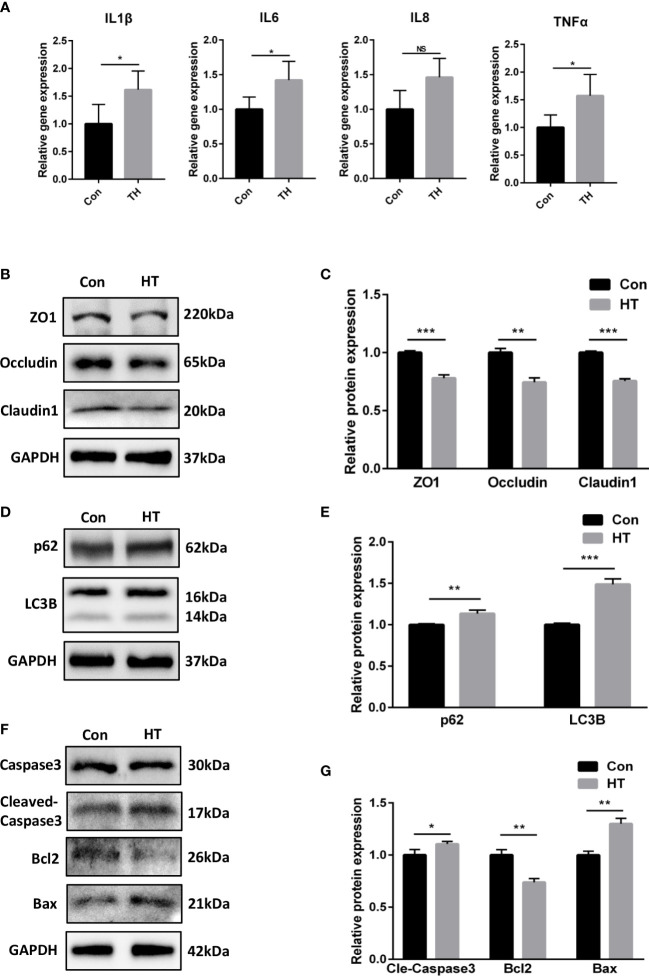
Homocysteine thiolactone may play a role in the proinflammatory property of *Fn*. **(A)** Relative gene expression of IL-1β, IL-6, IL-8 and TNF-α in Caco-2 cells treated with HT. **(B, C)** Representative ZO-1, occludin and claudin1 western blots of Caco-2 cells treated with HT. and statistical analysis. **(D, E)** Representative LC3B and SQSTM1/p62 western blots of Caco-2 cells and statistical analysis. **(F, G)** Representative caspase3, cleaved-caspase3, bcl2 and bax western blots of Caco-2 cells and statistical analysis. *P<0.05, **P<0.01, ***P<0.001. NS, Not statistically significant.

Since we observed that fucose treatment altered the metabolism of *Fn*, and the supernatant of fucose-treated *Fn* triggered less inflammation in Caco-2 cells, we speculated that fucose may reduce the secretion of some proinflammatory metabolites of *Fn*. As mentioned above, the decreased metabolite HT was found to induce oxidative stress and apoptosis in retinal pigment epithelial cells (AnandBabu et al., 2019). We speculate that there may be similar effects in IECs. As a result, HT treatment increased the mRNA expression of inflammatory cytokines IL-1β, IL-6, and TNF-α in Caco-2 (**Figure 8A**). In addition, the protein expression of tight junction proteins ZO-1, occludin, and claudin1 was decreased, though not so severely (**Figures 8B, C**). Autophagy was blocked to some extent, as the expression of LC3B-II and p62 was increased (**Figures 8D, E**). Meanwhile, the expression of bax, cleaved-caspase3, and bcl2 altered correspondingly, indicating apoptosis of Caco-2 cells after HT treatment (**Figures 8F, G**).

## 4 Discussion

In the herein study, fucose was discovered to ameliorate the proinflammatory property of *Fn* in colitis. Infection of fucose-treated *Fn* in mice that received DSS treatment resulted in less inflammatory cytokines release, tight junction damage, autophagy block, and apoptosis in IECs compared to *Fn*. Moreover, the metabolism of *Fn* was altered after fucose treatment. *In vitro* experiments revealed that the supernatant of fucose-treated *Fn* induced lower inflammatory damage in IECs compared to *Fn*, which demonstrated the role of metabolites alteration in fucose-induced proinflammatory property decreasing of *Fn* (**Supplementary Figure 1**).

Existing studies have reported the association between *Fn* and colitis. However, little was focused on the treatment strategy for *Fn*. Fucose was reported to play a critical role in sustaining gut homeostasis. Epithelial fucose was an important element of the intestinal barrier that protects the gut against pathogens and inhibits colonization of pathogen (Pham et al., 2014; Garber et al., 2021). Furthermore, exogenous fucose could alleviate DSS-induced colitis by regulating gut microbial structures and functions (Borisova et al., 2020; Ke et al., 2020). It is reported that fucose promotes the colonization of *Bifidobacterium* spp. and *Lactobacillus* spp., and promotes *Bifidobacterium*-related tryptophan metabolism in DSS-induced colitis (Li et al., 2019; Borisova et al., 2020). Notably, a recent study found that fucose influences the chemotaxis of *Campylobacter jejuni* and reduces its biofilm formation, indicating an inhibition effect of fucose on intestinal pathogen (Dwivedi et al., 2016). Similarly, our study found fucose addition during *Fn* culture could reduce the *Fn*-induced damage to intestinal epithelium under an inflammatory environment. All these studies support the role of fucose as a promising functional food in coordinating gut microbiome and inflammation.

Increasing studies explored the underlying mechanisms by which *Fn* aggravates the progression of colitis, including modulating the immune microenvironment, expanding myeloid-derived immune cells, activating the NF-κB inflammation pathway and so on (Kostic et al., 2013; Yang et al., 2017). Since we found that *Fn* disturbs autophagic flux in IECs in colitis previously (Duan et al., 2021), we wondered whether fucose treatment could reduce this ability of *Fn*. As expected, fucose-treated *Fn* induced less autophagy blockage. Autophagy disorder leads to cell apoptosis, and this is another way by which *Fn* contributes to colitis progression (Su et al., 2020). Fucose-treated *Fn* induced less cell apoptosis, which was compatible with prior results. These results further support the inhibition effects of fucose on the proinflammatory ability of *Fn*.

Why fucose treatment could decrease the proinflammatory characteristic of *Fn* is the next question. Many researches revealed the diverse roles of bacterial metabolism in the pathogenesis of colitis. For example, decreased gut microbiota metabolism of tryptophan leads to less aryl hydrocarbon receptor activation and intestinal inflammation exacerbation, *Eggerthella lenta* worsens colitis through metabolizing steroidal glycosides and driving Th17-dependent autoimmunity (Lamas et al., 2016; Alexander et al., 2022). Similarly, extracellular vesicles of *Fn*, which contain metabolites and bioactive proteins, were found to promote epithelial barrier damage and aggravate intestinal inflammation (Alexander et al., 2022). Since fucose could affect bacterial metabolism and reduces the expression of virulence genes (Pickard et al., 2014), we examined the metabolism of fucose-treated *Fn*. There indeed were changes in metabolic pathways such as biotin metabolism, histidine metabolism, riboflavin metabolism, and so on. Some proinflammatory metabolites such as D-glycero-D-manno-Heptose 1-phosphate, which could activate the NF-κB pathway and inflammatory cytokines production in colonic epithelial cells (Adekoya et al., 2018), and HT, which induces oxidative stress and apoptosis in retinal pigment epithelial cells (AnandBabu et al., 2019), were reduced in fucose-treated *Fn*. *In vitro* experiments demonstrated that the proinflammatory property of altered metabolites of *Fn* in IECs was decreased. We also examined the effects of HT on IECs and detect similar results with retinal pigment epithelial cells mentioned above. These results gave more evidence that fucose mitigates the proinflammatory property of *Fn*. Actually, there should be other metabolites that contribute to the inflammatory effect because the influence of HT was relatively small compared to the bacterial supernatant.

There were still some limitations in the current study. On the one hand, the specific mechanism that how fucose influences the metabolism of *Fn* and whether fucose affects the virulence genes expression at the same time was unknown. On the other, whether exogenous fucose could alter the metabolism of *Fn in vivo* equally and relieve *Fn-*related inflammation need further studies to explore. In addition, as a prebiotic, fucose may be fermented by other gut bacteria and influence other aspects of intestinal function. For instance, increasing the production of short-chain fatty acids, stimulating bacteria growth such as Escherichia coli, and affecting bowel habits such as defecation (Cummings et al., 2001; Kim et al., 2019). A recent study found that idiopathic chronic diarrhea macaques host produces fucosylated mucins that act as carbon sources or adhesion sites for potentially pathogenic microbes such as *Haemophilus* and *Campylobacter* (Westreich et al., 2019). Therefore, further studies are necessary to reveal the comprehensive effects or side effects of fucose on gut microbe and intestinal functions to support the appropriate usage of fucose.

In conclusion, our study demonstrated that fucose treatment reduces the proinflammatory effects of *Fn* in DSS-induced colitis and *in vitro* Caco-2 cells model. The underlying mechanism was to alter the metabolism of *Fn* and reduce the production of proinflammatory metabolites such as HT, thus decreasing apoptosis, autophagy blocking of IECs, and tight junction damage. These results may provide new insight into the application of fucose as a prebiotic in the treatment of *Fn*-related colitis and regulating the interaction between gut microbe and IECs.

## Data availability statement

The raw data supporting the conclusions of this article will be made available by the authors, without undue reservation.

## Ethics statement

The animal study was reviewed and approved by the Animal Experimentation Ethics Committee of Huazhong University of Science and Technology (Approval ID 2020-2529).

## Author contributions

CD performed the experiences, analyzed the data, and drafted the manuscript; LH performed a part of experiences and analyzed the data. JW and WQ supported the data evaluation; XD helped culture bacteria. CH and XH designed the study, revised the manuscript, provided funding, and obtained grant. All authors contributed to the article and approved the submitted version.
