# Supplementary figures and images for: Fucose ameliorates the proinflammatory property of Fusobacterium nucleatum in colitis via altering its metabolism
Source: Front Cell Infect Microbiol. 2023 May 1;13:1190602. doi: 10.3389/fcimb.2023.1190602 (PMC10183584; doi:10.3389/fcimb.2023.1190602)

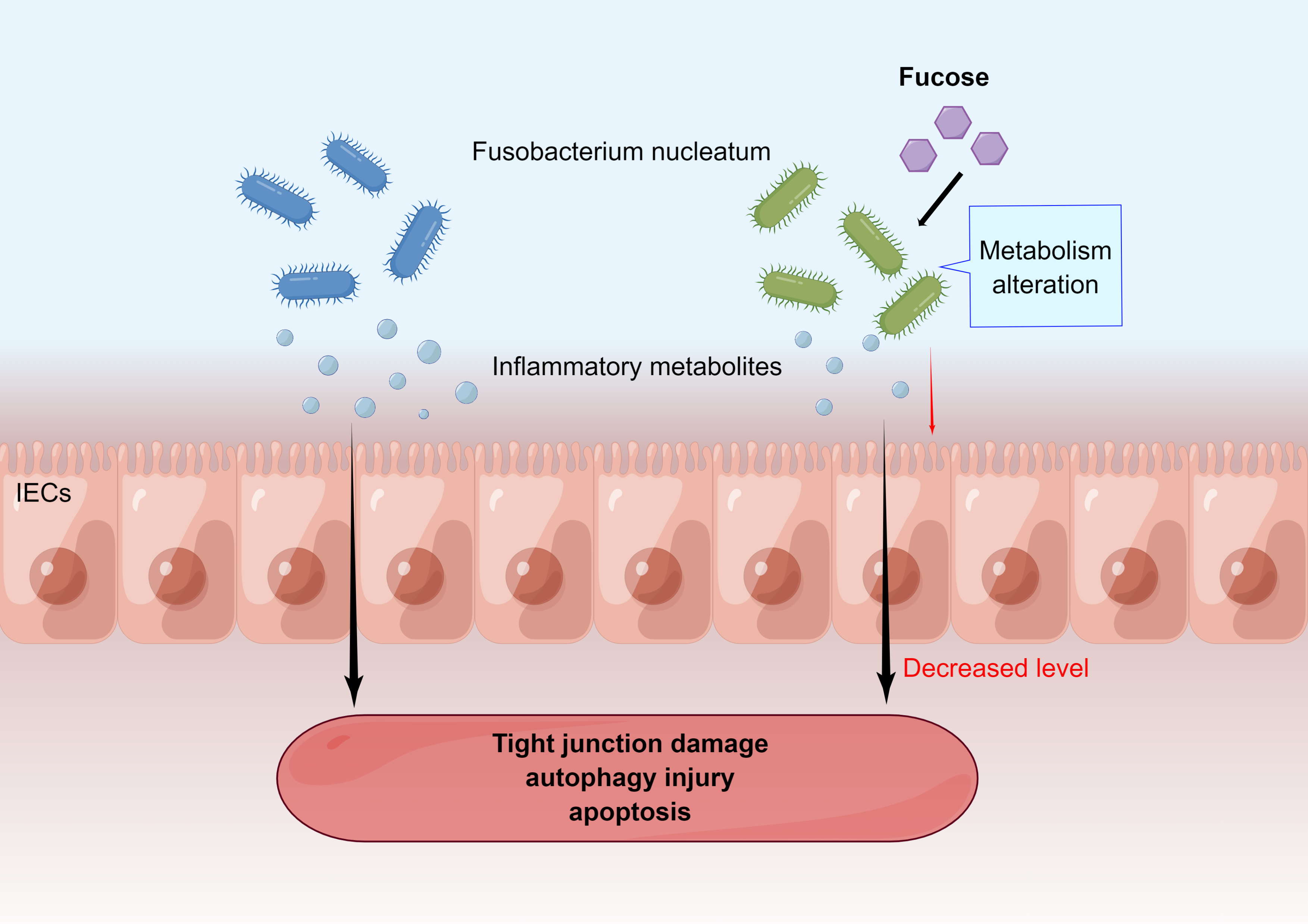

Supplement: Supplementary file 1 [file Image_1.tif]
